# RETRACTION: Upregulation of miR‐138 Increases Sensitivity to Cisplatin in Hepatocellular Carcinoma by Regulating EZH2

**DOI:** 10.1155/bmri/9875356

**Published:** 2026-07-31

**Authors:** BioMed Research International

RETRACTION: T. Zeng, L. Luo, Y. Huang, X. Ye, and J. Lin, “Upregulation of miR‐138 Increases Sensitivity to Cisplatin in Hepatocellular Carcinoma by Regulating EZH2,” *BioMed Research International* 2021, no. 1 (2021): 1‐12, https://doi.org/10.1155/2021/6665918.

The above article, published online on 8 March 2021 in Wiley Online Library (wileyonlinelibrary.com), has been retracted by agreement between the journal Editor‐in‐Chief, Yoel A. Klug; and John Wiley & Sons Ltd.

The retraction has been agreed following an investigation into concerns initially raised on PubPeer [1]. Substantial similarities were identified between the vimentin Western blots in the HepG2 human cell line panel of Figure 6B, and the Trib3 Western blots in Figures 6 L and 6 N of a published research article by a different author group involving cells obtained from mice and different experimental targets [2].

The authors’ explanation and supporting documentation did not adequately address our concerns. As a result, the data and conclusions of this article are considered unreliable; therefore, the article is retracted.

The authors did not respond when they were informed of the decision to retract.
